# Evaluating maternity care using national administrative health datasets: How are statistics affected by the quality of data on method of delivery?

**DOI:** 10.1186/1472-6963-13-200

**Published:** 2013-05-30

**Authors:** Hannah E Knight, Ipek Gurol-Urganci, Tahir A Mahmood, Allan Templeton, David Richmond, Jan H van der Meulen, David A Cromwell

**Affiliations:** 1 Office for Research and Clinical Audit, Lindsay Stewart R&D Centre, Royal College of Obstetricians and Gynaecologists, London 4RG, NW1, UK; 2 Department of Health Services Research and Policy, London School of Hygiene and Tropical Medicine, London, WC1H 9SH, UK

**Keywords:** Administrative health data, Maternity statistics, Method of delivery, Procedure codes, HES

## Abstract

**Background:**

Information on maternity services is increasingly derived from national administrative health data. We evaluated how statistics on maternity care in England were affected by the completeness and consistency of data on “method of delivery” in a national dataset.

**Methods:**

Singleton deliveries occurring between April 2009 and March 2010 in English NHS trusts were extracted from the Hospital Episode Statistics (HES) database. In HES, method of delivery can be entered twice: 1) as a procedure code in core fields, and 2) in supplementary maternity fields. We examined overall consistency of these data sources at a national level and among individual trusts. The impact of different analysis rules for handling inconsistent data was then examined using three maternity statistics: emergency caesarean section (CS) rate; third/fourth degree tear rate amongst instrumental deliveries, and elective CS rate for breech presentation.

**Results:**

We identified 629,049 singleton deliveries. Method of delivery was not entered as a procedure or in the supplementary fields in 0.8% and 12.5% of records, respectively. In 545,594 records containing both data items, method of delivery was coded consistently in 96.3% (kappa = 0.93; p < 0.001). Eleven of 136 NHS trusts had comparatively poor consistency (<92%) suggesting systematic data entry errors. The different analysis rules had a small effect on the statistics at a national level but the effect could be substantial for individual NHS trusts. The elective CS rate for breech was most sensitive to the chosen analysis rule.

**Conclusions:**

Organisational maternity statistics are sensitive to inconsistencies in data on method of delivery, and publications of quality indicators should describe how such data were handled. Overall, method of delivery is coded consistently in English administrative health data.

## Background

Countries which have administrative health data collection systems are increasingly using this information to produce maternity statistics at both local and national levels [1-3]. In the US, the Agency for Healthcare Research and Quality (AHRQ) developed a set of quality indicators based on administrative health data which included several areas of obstetric care [2]. These indicators have supported both national and local quality initiatives, and have been piloted in other developed countries including the UK, Canada, Spain, and Australia [4]. However, data quality remains a key concern for users of administrative maternity data and validation exercises are required to determine its accuracy and reliability prior to analysis [5].

In England, maternity statistics are produced by a number of organisations using the Hospital Episode Statistics (HES) database [6-9]. HES contains records on all patients admitted to English NHS hospitals, with data being extracted from local patient administration systems. The core fields of a HES record hold data on patient demographics and can capture up to 20 diagnoses and 24 procedures per episode of care. Delivery records can also capture supplementary data on the pregnancy and delivery, such as length of gestation, onset of labour, method of delivery and birth weight, for up to 9 babies. Not all delivery records contain this supplementary information (the ‘maternity tail’), although the percentage of records with a complete maternity tail has improved over time.

A number of quality indicators for hospital maternity services require method of delivery for their construction, for example the caesarean section rate (where it is required for the numerator), and the rate of third/fourth degree perineal tears amongst women delivering vaginally (where it is used in the denominator). Despite the importance of data on method of delivery, there is no current information on the quality of this data in HES. This is of concern because there are two ways in which method of delivery can be recorded in HES, and it is not clear which is the preferred data source. Until 2006, the UK Department of Health published figures on the consistency of the two sources of method of delivery for each hospital [10]. In addition, the Department of Health used to conduct extensive cleaning of HES data before its release for secondary analysis, but this has been replaced by a simpler data validation process.

This paper describes an evaluation of how statistics on maternity care in English hospitals are affected by the completeness and consistency of data on method of delivery. The completeness and internal consistency of HES method of delivery data were evaluated at a national level and by NHS trust (hospital organisation). We then assessed how different analysis rules for handling poor quality HES data influenced a selection of maternity statistics.

## Methods

We extracted from the HES database records of women who delivered in English NHS acute trusts between 1 April 2009 and 31 March 2010. Records were defined as relating to a delivery if “method of delivery” information was found in any procedure field and/or the maternity tail field. Table 1 maps the Office of Population Census and Surveys (OPCS) procedure codes R17-25 on to the maternity tail codes used to define method of delivery [11]. The definitions for each category are equivalent. However, the OPCS codes are entered by clinical coders based on the discharge notes, whereas the maternity tail data is typically populated directly from the electronic maternity information system, which is completed by midwives.

**Table 1:** **Correspondence between OPCS procedure delivery codes and maternity tail** “**delmeth**” **delivery codes**

**OPCS code**	**Delmeth code**	**Method of delivery description**
R17	7	Elective caesarean section
R18, R25.1	8	Emergency caesarean section
R19, R20	5, 6	Breech vaginal delivery
R21	2, 3	Forceps delivery
R22	4	Vacuum delivery
R23, R24	0, 1	Cephalic vaginal delivery without instruments
R25.2, R25.8, R25.9	9	Other method of delivery, including destructive operation to facilitate delivery

Both coding systems define elective caesareans as prelabour caesarean sections and emergency caesareans as intrapartum caesarean sections.

The analysis was limited to singleton deliveries. Records were excluded if they contained an International Classification of Diseases (ICD-10) diagnosis code for a multiple delivery (O30.1, Z37.2-.7 or Z38.3-.8) in any diagnosis field or the record contained data on more than one baby in the maternity tail.

Method of delivery was defined using a seven-category classification (Table 1). Both OPCS and maternity tail coding systems define ‘elective caesareans’ as prelabour caesarean sections and ‘emergency caesareans’ as an intrapartum caesarean sections. On inspection, a small number of hospitals had a value of “9” (other) in the maternity tail field for *all* deliveries, or seemed to have used this code to indicate an ‘unknown’ method of delivery. Consequently, if an NHS trust had values of “9” in the maternity tail field for more than 5% of their delivery episodes, all these values was re-coded as missing.

### Data analysis

To examine data completeness for each NHS trust, we calculated the proportion of women for whom the method of delivery was recorded in a) the procedure fields, and b) the maternity tail. This analysis included all singleton delivery records. The subsequent analysis of coding consistency was restricted to women whose records contained information on method of delivery in both sources.

The mean rate of coding consistency was calculated by dividing the number of records with a consistent mode of delivery recorded in both the procedure field and the maternity tail by the total number of records containing valid information in both fields. We measured the overall level of coding agreement at a national level using the unweighted kappa (k) statistic. This measure ranges from 0 (a level of agreement no greater than would be obtained by chance) to 1 (perfect agreement). Values of k above 0.80 are generally considered to indicate excellent agreement [12].

We used funnel plots to examine variation among NHS trusts in the consistency of method of delivery coding [13,14]. The inner and outer control limits set at two and three standard deviations above and below the national average, respectively. The limits also took into account a measure of over-dispersion. This was derived using the random-effects method and incorporated 10% winsorisation to prevent the limits being widened excessively by extreme outliers [14]. The 0-5th percentiles were winsorised to the 5th percentile and the 95-100th percentiles were winsorised to the 95th percentiles.

We selected three maternity statistics to investigate the impact of using different analysis rules for handling inconsistent data. These were selected to represent the various categories of maternity statistic that require method of delivery:

•Emergency caesarean section rate (where method of delivery is the numerator);

•Third and fourth degree perineal tear rate amongst instrumental deliveries (where method of delivery is the denominator), and

•Elective caesarean section rate for breech presentation (where method of delivery affects both numerator and denominator).

Breech presentation was defined using ICD-10 codes O32.1, O64.1, O80.1 and O83.0-1 and/or OPCS or maternity tail codes for breech vaginal deliveries. We defined third and fourth degree tears as records with an ICD-10 code for third or fourth degree perineal laceration (O70.1; O70.2) and/or an OPCS procedure code for their repair (R32.1; R32.2).

Five versions of each statistic were produced using different analysis rules for dealing with inconsistencies in the method of delivery data (see Table 2 for definitions). To investigate the impact of these different rules on trust-level maternity statistics, we used mean-difference plots [15] to assess the agreement between two sets of figures, namely, figures derived using data on method of delivery from only the procedure fields (the approach currently used by the NHS Information Centre) [10] and figures derived using only those records for which the procedure and maternity tail data were in agreement (the most restrictive of the five analysis rules). STATA 11 (TX: StataCorp LP) was used for all statistical calculations.

**Table 2:** **Impact of mode of delivery definition on resulting maternity statistics**: **three case studies**

**Definition rule**	**Numerator**	**Denominator**	**# Trusts**	**Rate (%)**
**Emergency caesarean section rate**				
1	89,572	624,199	151	14.35
2	75,370	550,763	140	13.68
3	70,298	525,192	140	13.39
4	81,964	573,497	140	14.29
5	82,701	578,223	140	14.30
**Third and fourth degree perineal tear rate amongst instrumental deliveries**				
1	5,049	76,161	144	6.63
2	4,260	64,661	136	6.59
3	3,965	60,281	135	6.58
4	4,640	70,268	133	6.60
5	4,686	71,205	133	5.58
**Elective caesarean section rate for breech presentation**				
1	11,852	23,640	147	50.14
2	10,066	21,691	139	46.41
3	9,378	17,834	138	52.58
4	10,919	21,776	136	50.14
5	10,928	21,851	136	50.01

Key to definition rules.

1 = Use all episodes with an OPCS method of delivery code & base method of delivery definitions on OPCS codes alone.

2 = Use all episodes with a delmeth code & base method of delivery definitions on delmeth codes alone.

3 = Use only episodes in which both OPCS and delmeth codes are present and in agreement; base method of delivery definitions on agreed method of delivery code.

4 = Use all episodes with an OPCS method of delivery code excluding those from trusts with a poor agreement rate; base method of delivery definitions on OPCS codes alone.

5 = Use all episodes excluding those from trusts with a poor agreement rate; base method of delivery definitions on OPCS codes, or delmeth codes if OPCS codes are missing.

## Results

### Completeness of method of delivery codes

**Figure 1:**
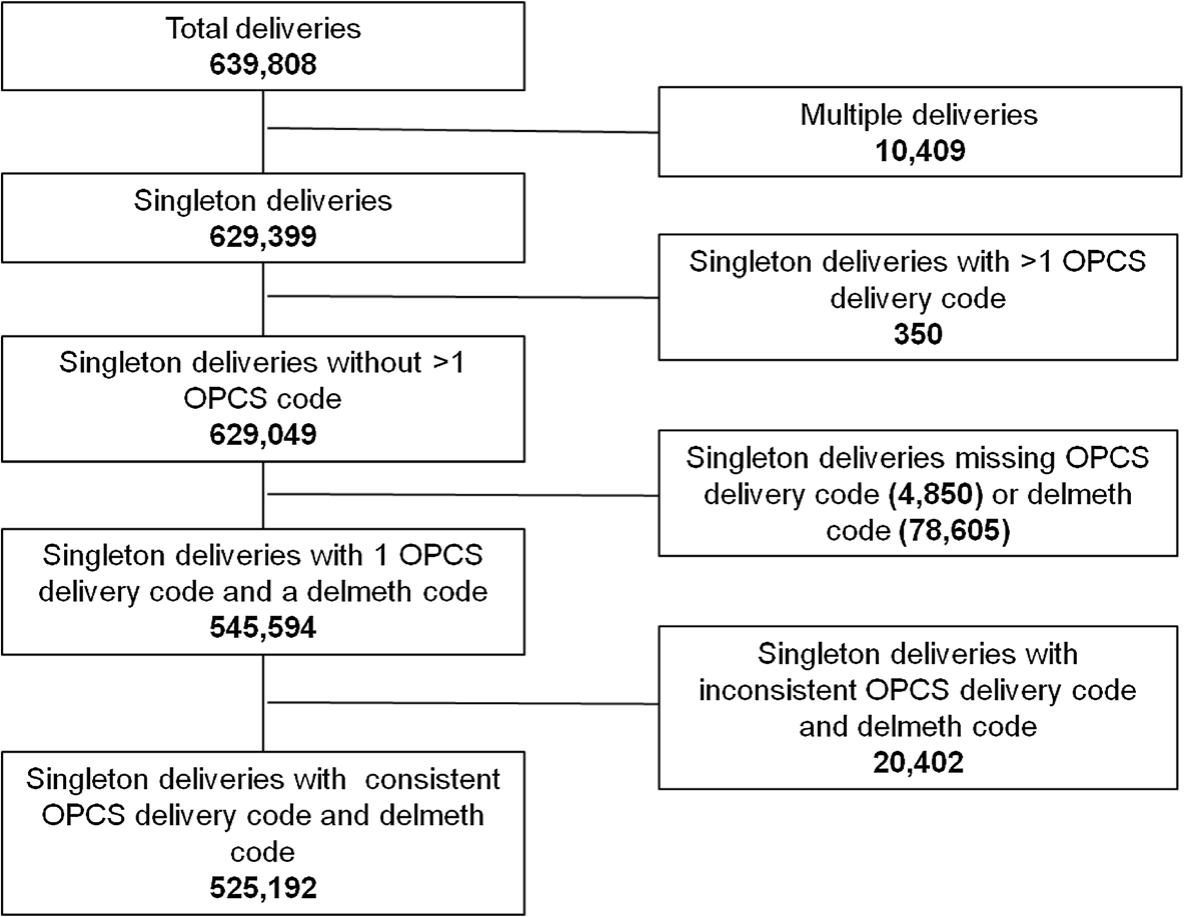
Flow chart.

We identified 629,049 singleton deliveries in 151 English NHS trusts between 1 April 2009 and 31 March 2010. Among these, 545,594 records (86.7%) had method of delivery entered in both the procedure and maternity tail fields (Figure 1).

Method of delivery was mostly commonly entered as a procedure code, being omitted in just 4,850 records (0.8%) overall. All but four NHS trusts had a “method of delivery” procedure code in more than 95% of their deliveries, and in no trust was this code available in less than 90% of deliveries. In contrast, 78,605 records (12.5%) had method of delivery missing from the maternity tail. Only 96 of the 151 NHS trusts had a maternity tail “delivery” code in more than 95% of their deliveries, and seven NHS trusts had no information on delivery method in the maternity tail of their records.

### Overall coding consistency

Among the 545,594 singleton deliveries with information in both the procedure and maternity tail fields, method of delivery was coded consistently in 96.3% records (kappa = 0.93, p < 0.001) using the seven category coding framework (Table 3). The overall rate of each delivery method differed by between 0 and 0.5% (e.g. the overall emergency caesarean section rate was 13.9% (76,004/545,594) from procedure codes and 13.7% (74,539/545,594) from maternity tail codes). However, coding inconsistencies had a large relative effect on the proportion of breech vaginal deliveries because it was an uncommon method of delivery. There were nearly twice as many records having this method of delivery in the maternity tail (3,170) compared to the procedures field (1,809) (Table 3).

**Table 3:** **Consistency of Method of delivery in English NHS trusts in 2009**/**10 as defined using OPCS delivery code and the maternity tail delmeth code**

**Method of delivery(OPCS)**								
**Method of Delivery(Delmeth)**	**Elective CS**	**Emergency CS**	**Breech vaginal**	**Forceps**	**Vacuum**	**Cephalic vaginal**	**Other**	**Row total**
Elective CS	**47**,**623**	4,131	15	26	186	139	1	52,121
Emergency CS	3,890	**70**,**298**	18	17	29	287	0	74,539
Breech vaginal	101	115	**1**,**547**	63	919	424	1	3,170
Forceps	324	173	5	**28**,**755**	438	414	0	30,109
Vacuum	13	52	3	1,347	**31**,**526**	493	0	33,422
Cephalic vaginal	396	904	202	1,573	1,481	**345**,**723**	37	350,316
Other	125	332	19	727	50	652	**1**	1,906
Total	52,472	76,004	1,809	32,508	34,629	348,132	40	545,594

Among all coding disagreements, 39% were inconsistencies between elective and emergency caesarean section (=[4,131 + 3,890]/20,402), while 19% were inconsistencies between instrumental and non-instrumental vaginal delivery (= [1,573 + 1,481 + 414 + 493]/20,402). A further 9% of inconsistencies were related to the type of instrument used to assist the delivery of the baby (=[1,573 + 1,481 + 414 + 493]/20,402) (see Table 3).

### Variation in coding consistency between NHS hospital trusts

**Figure 2:**
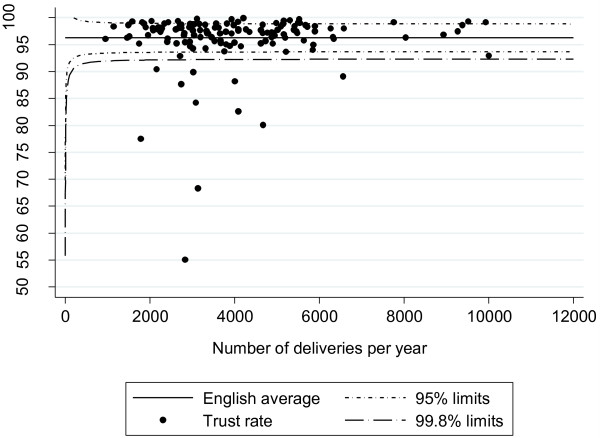
**Funnel plot showing consistency between OPCS mode of delivery code and delmeth code for English NHS trusts (2009/10).** The English average was calculated by dividing the number of records with consistent mode of delivery recorded in both fields by the total number of records containing information about mode of delivery in both fields.

Figure 2 shows the variation in coding consistency among the 136 NHS trusts that had more than 500 delivery records containing both procedure and maternity tail codes. Eleven NHS trusts had levels of coding consistency lower than 92%, which was poorer performance than would be expected from random variation alone. The coding inconsistencies in these trusts appeared to be occurring systematically, accounting for 31% of all emergency/elective caesarean section discrepancies, 42% of all forceps/vacuum delivery discrepancies, 28% of all instrumental/non-instrumental delivery discrepancies, and 99% of all breech/vacuum delivery discrepancies.

The 11 NHS trusts with “poor” data quality accounted for 38,100 (7%) of the 545,594 singleton deliveries. Removing these trusts from the analysis improved the overall level of coding agreement from 96.3% (kappa = 0.93, p < 0.001) to 97.4% (kappa = 0.95, p < 0.001).

### Impact of rules for handling data inconsistencies on maternity statistics

Table 2 shows the impact of using different analysis rules upon the three selected maternity statistics. At a national level, the different definitions had the smallest impact on the overall rate of third/fourth degree perineal tears amongst instrumental deliveries, with only 0.05% difference between the lowest and highest estimates. For the emergency caesarean section rate, the difference was almost 1%.

The most unstable statistic was the elective caesarean section rate among all women with breech presentation, with the estimated proportion ranging between 46.4% and 52.6% depending on which analysis rule was used. The inconsistencies in the definition of elective caesarean section affected the numerator, while the denominator was affected by the poor consistency in the definition of breech delivery.

**Figure 3:**
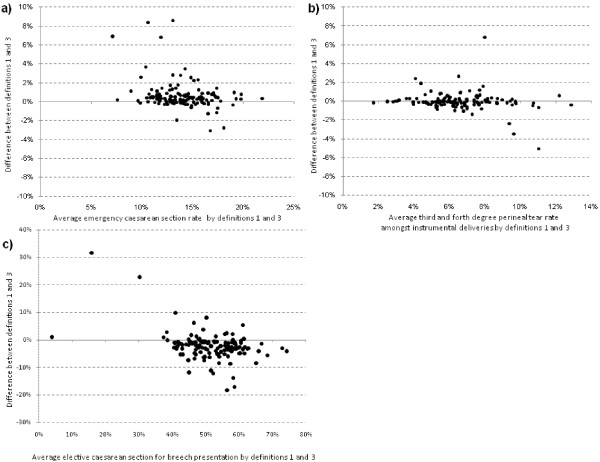
**Mean difference plots: Impact of using different method of delivery definitions on trust-level rates of : a) emergency caesarean section; b) third and fourth degree perineal tears amongst instrumental deliveries, and c) elective caesarean section for breech presentation.** Definition 1: Use all episodes with an OPCS method of delivery code & base method of delivery definitions on OPCS codes alone; Definition 3: Uses only episodes in which both OPCS and maternity tail codes are present and in agreement; base method of delivery definitions on agreed method of delivery code.

Figure 3 shows the difference between the figures derived using two analysis rules at the level of individual NHS trusts. For the majority of NHS trusts, there was little difference between the emergency caesarean section rate and the third/fourth degree perineal tear rate amongst instrumental deliveries. The standard deviation (SD) of the difference was 1.6% and 1.0%, respectively. The spread of differences was larger for the elective caesarean section rate for breech presentation, with the SD of the differences being 5.5%. This reflects its comparatively smaller sample size in relation to the other statistics. Further analysis of these results showed that, for most trusts, the differences arose from changes in hospital sample size due to incomplete maternity tail data rather than inconsistencies of coding (Figure 2). Nonetheless, for each statistic, the different analysis rules produced very different figures for some NHS trusts, and these were typically those with poorer levels of coding consistency.

## Discussion and conclusion

This study evaluated the completeness and internal consistency of data on method of delivery within the HES database and how the accuracy of this data could affect different maternity statistics. We found that the procedure fields contained the most complete information on method of delivery, being available in 99.2% of records. They were also more consistently complete across all NHS trusts. The completeness of maternity tail information was considerably lower, and was missing entirely for seven NHS trusts.

When information was available in both sources, there was a high level of agreement between the method of delivery codes overall. Inconsistent coding was a problem in a minority of NHS trusts, with only 11 out of 136 trusts showing divergent coding practices. It was, therefore, not surprising that, at a national level, different rules for handling inconsistent data had a small effect on the derived statistics. Nonetheless, the degree of sensitivity varied across the statistics tested.

The variation in the level of data completeness and coding consistencies across NHS trusts meant that, for all statistics tested, the differences in the estimates produced by the alternative analysis rules were substantial for some trusts. These results highlight the need for a careful assessment of data quality and for the transparent reporting of how incomplete and inconsistent data are handled when producing maternity statistics, particularly at an organisational level.

This study included all singleton deliveries occurring in English NHS maternity units, providing a very large sample size for analysis and thereby reducing the risk of selection bias. We identified 629,049 singleton deliveries during the study time period, which represents approximately 97% of all hospital deliveries registered in England during 2009/10 by the Office for National Statistics [16]. Previous research shows that women with severe morbidity and prolonged hospitalisation are more likely to have delivery information missing from their records [17]. Although the loss of these women from analyses of mode of delivery is unlikely to make a difference, it would become extremely important if the data are used to assess maternal or perinatal morbidity and mortality.

A limitation of this evaluation is that it only assessed internal consistency. We did not attempt to validate the HES dataset by comparing a sample of records against hospital medical records. We are not aware of any studies that have specifically validated “method of delivery” coding in HES against hospital records, but studies of similar administrative health databases in other countries have reported high levels of agreement (kappa > 0.98, where stated) [18-21].

The seven method of delivery categories used in this study represent only one possible classification. The grouping was dictated by the OPCS procedure and maternity tail codes. A weakness of this classification is the definition of caesarean section as either elective or emergency. The 2004 NICE guideline recommended that the urgency of a caesarean section be indicated using the Lucas/National Confidential Enquiry into Patient Outcome and Death (NCEPOD) classification and noted that replacing the terms ‘emergency’ and ‘elective’ with its four grades of urgency would aid communication between health professionals [22]. Currently, the HES database is unable to capture this classification system.

Data quality is a concern for healthcare providers, managers and policy makers [23]. In England, the Care Quality Commission now mandates an annual audit of data quality within NHS trusts, [24] and a recent systematic review of coding accuracy in all types of routinely collected hospital discharge data found that coding accuracy rates have been improving [25]. Since 2002, the coding of primary diagnosis within HES has improved in accuracy from 73.8 per to 96.0% when compared against case notes [24].

The results of this study add to this work by addressing concerns about the quality of HES maternity data [26]. The high level of consistency in the recording of method of delivery overall supports its use for the construction of national maternity statistics. Coding disagreements were most common for the categories of emergency and elective caesarean section. Nonetheless, overall consistency was excellent between both emergency (kappa = 0.92; p < 0.001) and elective (kappa = 0.90; p < 0.001) caesarean section procedure and maternity tail codes. This supports a previous conclusion that coding errors were unlikely to account for the large variation in the rates of emergency caesarean section observed between NHS trusts [27].

At an NHS trust level, levels of consistency were high for the majority of organisations, which provides evidence to support the use of HES-based quality indicators for the purpose of comparing the performance of NHS trusts. However, our results illustrate the importance of addressing data quality within NHS trusts with divergent coding practices. The risk of organisations being mistakenly identified as “outliers” on performance indicators due to data errors is well-known. Our results suggest this risk is also increased by the sensitivity of maternity statistics to the analysis rules used to handle inconsistent data.

The study’s results also suggest that any publishers of maternity statistics should describe details of how data quality was assessed and incomplete and consistent data were handled in the analysis. In England, the Health and Social Care Information Centre (HSCIC) publishes maternity statistics at Strategic Health Authority, NHS trust and individual unit level annually [3]. This public body is England's central source of health and social care information and the value of its publications on maternity services would be enhanced if they again provided information on the level of agreement between data in the procedure fields and in the maternity tail.

Providing methodological information may be more problematic for commercial companies that supply hospitals with comparative measures of organisational performance given the need to balance transparency with the protection of intellectual property. Nonetheless, companies that provide maternity benchmarking services could be required to meet minimum standards of transparency as part of the conditions of access to administrative health data. Whilst national trends and local over time can be reported as long as the definitions used by these organisations remain the same, the definitions used are still important for interpretation.

## Implications

Approaches to validate the use of administrative health data for maternity statistics commonly fall into two categories. They either check the consistency of the administrative health data against medical records [17-20,28] or against another source of maternity data such as national birth registers [29-31]. Such external validation studies can be time consuming, costly and technically challenging, as well as raising ethical and information governance issues related to access and data linkage. We used a particular feature of HES to examine its internal consistency and this is an example of how relationships within administrative health data can be used to identify organisations with divergent coding practices [32]. Whilst external validation should remain the “gold standard”, this approach to data quality assessment is simple to perform and has the potential to be developed more widely as a complementary technique.

## Abbreviations

HES: Hospital Episode Statistics; ARHQ: Agency for Healthcare Research and Quality; IC: Information Centre; NHS: National Health Service; OPCS: Office of Population Census and Surveys; ICD-10: International Classification of Diseases, 10th Edition.

## Competing interests

The authors declare that they have no competing interests.

## Authors’ contributions

DC, JvdM, TM, DR and AT conceived the idea. HK, DC, IG-U and JvdM designed the methodology. HK, IG-U and DC conducted the statistical analysis. HK wrote the manuscript. DC, JvdM, IG-U, TM, DR and AT commented on subsequent drafts and approved the final version. All authors read and approved the final manuscript.

## Pre-publication history

The pre-publication history for this paper can be accessed here:

http://www.biomedcentral.com/1472-6963/13/200/prepub
